# A narrative review on pressure ulcer (PU) studies relevant to medical imaging

**DOI:** 10.11604/pamj.2020.36.66.19431

**Published:** 2020-06-04

**Authors:** Seth Kwadjo Angmorterh, Andrew England, Judith Anaman-Torgbor, Nii Korley Kortei, Huseini Alidu, Cosmos Yarfi, Jo Webb, Eric Kwasi Ofori, Peter Hogg

**Affiliations:** 1 Department of Medical Imaging, School of Allied Health Sciences, University of Health and Allied Sciences (UHAS), Ho, Ghana,; 2 Directorate of Radiography, School of Healthcare Sciences, Allerton Building, University of Salford, Salford, Greater Manchester, United Kingdom,; 3 School of Nursing and Midwifery, University of Health and Allied Sciences (UHAS), Ho, Ghana,; 4 Department of Nutrition and Dietetics, School of Allied Health Sciences, University of Health and Allied Sciences (UHAS), Ho, Ghana,; 5 Department of Medical Laboratory Sciences, School of Allied Health Sciences, University of Health and Allied Sciences (UHAS), Ho, Ghana,; 6 Department of Physiotherapy and Rehabilitation Sciences, School of Allied Health Sciences, University of Health and Allied Sciences (UHAS), Ho, Ghana,; 7 Directorate of Occupational Therapy, School of Health Sciences, University of Salford, Salford, Greater Manchester, United Kingdom

**Keywords:** Pressure ulcers, interface pressure, radiography, tissue viability

## Abstract

Pressure ulcers (PUs) are defined as localised injuries to the skin and/or underlying tissue as a result of pressure or pressure together with shear. PUs present significant health implications to patients; costing billions to manage and/or treat. The burden of PU prevention in hospitals must be the concern of all healthcare professionals, including radiographers. The purpose of this narrative review article was to identify and critically evaluate relevant literature and research conducted into pressure ulcers (PUs) relevant to medical imaging. It is expected that this review article will increase the level of awareness about PUs amongst radiographers and help to develop appropriate interventions to minimise the risk of PUs. A literature search was conducted in PubMed/Medline, Scopus, CINAHL, and Google Scholar to retrieve relevant articles. Also, books, professional body guidelines, magazines, grey and unpublished literatures were also searched. The search was limited to English Language articles. Only five articles were retrieved and reviewed. There are limited studies on PUs relevant to medical imaging. Available studies provide some evidence that radiographic procedures and settings subject patients attending for radiographic procedures to the risk of PUs. Further studies are needed into PU risk assessment, minimisation and management in medical imaging to help raise awareness and address the problem of the potential for PU development.

## Introduction

Pressure ulcers (PUs) are defined as localised injuries to the skin and/or underlying tissue as a result of pressure or pressure together with shear [1] and can be categorised into six different categories; category one, two, three, four, unstageable/unclassified, and suspected deep tissue injury [2]. Enormous efforts have been directed at reducing the incidence of PUs among patients worldwide [2]. Notwithstanding, the occurrence of hospital-acquired (nosocomial) PUs continue to rise, accounting for significant complications and patient deaths [1]. PU risk assessment scales (RASs) are non-invasive, cost-effective, preventive tools for assessing patients´ risk of developing PUs [2]. Three main PU RASs exist; these are the Norton, Braden and Waterlow [2]. Patients´ risk of developing PUs is assessed by establishing an aggregate score according to a set of parameters, deemed to be risk factors. The prevalence of PUs varies across countries and across clinical settings. For example, the prevalence of PUs in the United Kingdom (UK) is 4.7% in care homes whereas the prevalence of PUs across hospitals in the United States (US), Canada and Nigeria is 12.3%, 13.8% and 36.8% respectively [3-6]. Prevalence data may not be the appropriate measure or indicator of the quality of care patients receive within a healthcare setting because different healthcare settings have patients with different health conditions, and at varying PU risk levels. PUs most commonly occur at the head, sacrum and heels, often referred to as the jeopardy areas, due to the prominent bony features at these anatomical areas [1, 2]. PUs have huge financial implications for healthcare providers, costing billions to treat and/or manage [7]. In addition to the financial costs, PUs have significant negative consequences on patients, impacting quality of life in terms of physical, psychological and social functioning [8].

Factors contributing to the formation of PUs include fat/muscle ratio, the tendons and ligaments involved, and the magnitude and duration of interface pressure (IP) [9-11]. IP is defined as the pressure between the human body and a supporting surface [12]. There are various benchmarks and durations above which IP may result in partial or complete occlusion of blood flow within the capillaries, thereby inducing the formation of PUs. Studies have indicated that an IP of 60 mmHg, sustained for a period of 60 minutes, may induce soft tissue damage and may lead to the development of PUs [9, 10]. Another study suggested that IPs between 32-47 mmHg may induce PUs formation [11]. The key point is that a lower IP sustained for a longer period is likely to cause as much harm as a high IP sustained for a short period. To reduce the risk of PU formation, the use of safe patient repositioning techniques and IP redistributing overlays, cushions and mattresses are standard practice in many healthcare settings. In radiography, previous studies [13, 14] have shown that medical device-related PUs may occur as a result of the direct contact with medical devices, and the presence of high IPs. The duration and some of the techniques applied during some radiographic procedures may increase the risk of PUs among patients attending for radiographic examinations. For example, during intravenous urography procedures, an abdominal compression band may be applied tightly across the lower abdomen to concentrate the contrast and fill the ureters and renal pelvis. The application of the compression band would increase the IP between the patient and the imaging table surface [14]. Confounding this, patients would have to remain in the compressed position for several minutes, sometimes up to 45 minutes depending on the clinical history and the specific needs of the patient. The clinical implication is that patients accessing prolonged radiographic procedures, such as intravenous urography, could be exposed to the risk of PU formation and the risk of PU is further compounded because pressure damage may take time to appear. This suggests that radiologists and radiographers may be unaware of the PUs formations due to prolonged radiographic procedures. The prevention of PUs should be based on a detailed understanding of predisposing/risk factors. The identification of patients at risk is a key component for the effective prevention of PUs across all healthcare settings. The burden of PUs in hospitals must be the concern of all healthcare professionals. Therefore, the purpose of this article is to identify and critically evaluate relevant literature and research conducted into the risk of PU development relevant to medical imaging. It is expected that this review article will increase the level of awareness about PUs amongst radiographers and help to develop appropriate interventions to minimise the risk of PUs.

## Methods

A literature search was conducted in PubMed/Medline, Scopus, CINAHL, and Google Scholar to retrieve relevant articles. Grey and unpublished literatures were also searched as well as books, professional body guidelines, magazines, and leaflets. The following keywords were used for the search: pressure ulcers, pressure sores, decubitus pressures, interface pressure, radiography, medical imaging, diagnostic imaging, and radiology. The Boolean operators AND and OR were used to improve the search. The search was limited to English Language articles. There was no initial date restriction on the search, but it ended in November, 2018. This is to ensure that seminal studies conducted many years ago and current literature were also captured in the search results. The narrative review method was chosen since this article sought to evaluate, summarise, and clarify literature on the risk of PUs development relevant to radiographic procedures and settings.

## Current status of knowledge

Five articles were retrieved and reviewed accordingly. A summary of the objectives, methods used, major findings and limitations of the articles are presented in Table 1. Angmorterh *et al*. [14] investigated the risk of PU, comfort, and pain among healthy volunteers in a MI environment. The authors used a calibrated XSENSOR™ pressure mapping system/software (version 6) to measure IP across three jeopardy areas (head, sacrum, and heels) on an X-ray table surface with no mattress, an X-ray table surface with a thin radiolucent mattress, and a curved computed tomography (CT) table surface with a thin mattress. To account for different ethnicities, ages, and body mass indices (BMIs), a disproportionate stratified random sampling method was used to recruit 46 volunteers aged 18-59 years. Each volunteer wore loose fitting leggings and a T-shirt and were asked to lie on the pressure mat in a supine position with the hands pronated and the hips adjusted to ensure that they were equidistant from the edges of the mat. The leggings were close fitting with four-way stretch to avoid wrinkling of fabric producing false areas of high pressure. Following a six-minute settling time, the volunteers were asked to remain still for 20 minutes whilst pressure mapping data were acquired after which an exit questionnaire on pain and comfort was administered to the volunteers to document their experiences. The exit questionnaire consisted of five questions/statements - three closed-ended and two open-ended questions. Example question one: on a scale of 1-5, how comfortable were you when lying on the medical MI table surface?” Responses (1 = very uncomfortable; 2 = uncomfortable; 3 = passable; 4 = comfortable; 5 = very comfortable). Volunteers were asked to tick the box that applies to them. One of the open-ended questions asked the volunteers to indicate on a human diagram the anatomical area where they experienced pain. The other question sought to solicit volunteers´ comments or opinions on the overall experience of lying on the MI table surfaces.

**Table 1:** summary of the objectives, methods used, major findings and limitations of the five articles on IP or PU in medical imaging (MI)

Authors	Objective (O)/Method (M)	Major findings	Major limitations
Justham *et al*. [13]	(O) Investigated the IP experienced by healthy volunteers on (MI) table surfaces. (M) Used Talley Oxford Pressure Monitor (TPM) mark III to measure IP	There was potential of high risk of IPs on MI surfaces. PU risk may exist on MI table surfaces.	The cell matrix of the TPM system has poor spatial resolution due to wide spaces between sensors, some as much as 100 mm.
Angmorterh *et al*. [14]	(O) Assessed level of IP on MI table surfaces (M) XSENSOR™ mat and questionnaire	IP risk exists on X-ray tables with no mattress.	No patients; all the participants were healthy volunteers.
Messer and Groer [15]	(O) Validated a PU risk assessment and preventive intervention tool for adult patients (M) Qualitative review and retrospective analysis of hospital records	PU risk assessment and preventive intervention instrument can be used to accurately predict the individual PU risks for hospital ancillary procedures.	Large numbers of risk factors are involved and may be difficult to administer such tool within radiographic settings.
Brown [16]	(O) Investigated patients′ risk for PUs (M) Braden scale and a skin inspection	PU risk exists on MI table surfaces. 54% of the patients acquired category one PUs from tables used in an imaging department.	Unspecified type of table surfaces used. Patient characteristics were not described.
Justham and Rolfe [17]	(O)Assessed radiography teachers′ knowledge about IP (M) Qualitative (interview)	Participants lacked knowledge on PU risk during radiographic procedures. Participants also lacked knowledge on measures to reduce the risk of PU in radiography.	Small sample size and the findings may not be a true reflection.

The results indicated that IPs of varying degrees exist on MI table surfaces. Analysis of variance identified statistically significant differences in the mean IP for the jeopardy areas across the three MI table surfaces (p ≤ 0.001) with the head registering the highest mean IP value (75.9 ± 6.9 mmHg) on the X-ray table without a mattress. The high levels of IP observed for the head indicated that tissue ischemia could be developed from lying (between 20-30 minutes) on radiological examination tables, and may in turn lead to PU formation in patients undergoing lengthy interventional radiology/radiography procedures. Seventy percent of the volunteers found lying on the X-ray table with no mattress to be very uncomfortable and sixty-seven percent experienced most pain whilst lying on the X-ray table with no mattress and over 81% of the pain was reported at the head. While the study conducted by Angmorterh *et al*. [14] provided some important information on PU risk, comfort, and pain in MI, the study focused on healthy volunteers who could lie still for 26 minutes without any difficulty unlike real life patients who may not be able to lie still for similar duration. In addition, those with weight greater than 250 kg and height more than 190 cm were excluded, leaving a wide knowledge gap on what the situation might be among patients and people with such characteristics. In the study [14], some evidence was adduced that BMI and mean IP for the whole body correlated and therefore knowledge of the effect of weight above 250 kg on IP will be very much valuable to the prevention of PUs but this was lacking. The study also failed to provide evidence on the level of PU risk, comfort, and pain among people with conditions such as back pain, scoliosis, or kyphosis as well as pregnant women. Regarding the measurement of IP on the MI tables, no analysis was conducted on a CT scanner with a flat table top (as in the case for radiotherapy planning), and MRI tables. In a retrospective study, Messer and Groer [15] validated a PU risk assessment tool for adult patients undergoing hospital diagnostic and treatment procedures. The author suggested that the PU risk assessment instrument can be used to accurately predict PU risk among patients attending for radiographic procedures. The first component of the study included a narrative literature review to investigate the relationships between extrinsic risk factors inherent in the hospital (pressure, friction, shear, temperature, and moisture), intrinsic factors (diabetes, neuropathy, and malnutrition), and risk of PUs. The second component of the study consisted of a quantitative analysis of the predictive power of selected risk factors based on logistic regression models and areas under the Receiver Operating Characteristic (ROC) curves. The dependent variable of interest was existing PU cases whereas the independent variables were potential intrinsic and extrinsic PU risk factors. PU risk factors identified included advanced age, Human Immunodeficiency Virus (HIV), diabetes, anaesthesia/sedation, and fever. An assessment scale was constructed and the accuracy of the scale was tested for generalisability and it was found to be an accurate predictor of the risk in this population, with a content validity of 0.91, indicating excellent validity. The PU risk assessment tool developed by Messer and Groer [15] may be useful in certain clinical settings (for example on hospital wards) but may be difficult to apply within the radiographic settings due to time constraints because it is made up of a large number of risk factors. Conventional MI procedures are normally performed within very short time frames (e.g. as little as five minutes) hence it is difficult to accommodate such an elaborate tool in conventional MI (e.g. projection radiography). Due to high workload and limited time to spend with each patient, radiographers may not have the time needed to risk assess patients with this risk assessment tool. Also, radiographers will require extensive training to be able to accurately use this risk assessment scale because PU risk assessments are not routinely performed within radiography departments.

In a prospective study by Brown [16], data were collected from 80 patients on four different mattresses/support surfaces used in an imaging department. Using the Braden PU RAS, each patient was assessed for risk of developing a PU, and the total score recorded. A skin inspection of eleven pressure areas pre and post-imaging were recorded, and the duration of the imaging examination was also documented. It is worth noting that the eleven pressure areas were not identified. Post examination skin inspection showed that 54% of the patients acquired category one PUs. The conclusion of the study is of concern, because it gives the indication that the risk of PU development may exist within the radiographic settings. The following study limitations have been observed. First, the study did not specify the types of mattress/support surfaces used. This is important because different support surfaces have different impacts on patient´s skin and some could be potential sources of tissue damage, hence it would have been very helpful if the researcher had indicated the type of support surfaces used for the experiment. For example, if the patients were made to lie on an X-ray table without a mattress or any form of cushion for a long time, then it would not be surprising that over half of the patients developed a category one PU. This is because such a surface is likely to increase patients´ IP, which may lead to an increased risk of developing PUs. Also, patient characteristics (e.g. health status, age, levels of nutrition and physical activity) were not reported. This is a significant limitation because studies have shown that the skin of older patients and those suffering from chronic diseases such as cancer are more prone to developing PUs [17]. In addition, none of the eleven areas inspected were named. Although the study concluded that more than half of the patients developed PUs, Brown [16] did not indicate the specific MI procedures they were referring to; hence the pressure injuries recorded in the study by Brown [16] cannot be attributed to the imaging surfaces because the study did not investigate the IPs experienced by these patients whilst lying on the imaging surfaces. The implication of this is that, the observed PUs might have arisen from a range of factors not related to the imaging process. Additionally, it is possible that this figure might have risen because studies have shown that skin damage due to pressure does not often appear on superficial tissues until at least three days post injury [17, 18].

Justham and Rolfe [19] investigated the level of knowledge on the risk, prevention and management of PUs among radiography teachers in the UK. A total of fourteen teachers participated in the study. In terms of work experience they were reported to have been involved in radiography up to approximately 30 years. They responded to four questions and one of the questions enquired about their views and experiences as radiographers. Thematic analysis was employed to analyse the data and the analysis provided a range of views on the risk of PUs development, length of procedures, position during procedure and the type of MI table. With regards to PU risk, the teachers noted that some patients, including the elderly, were at risk irrespective of the procedures being performed if the procedure lasted more than 10 minutes. According to the authors, irrespective of the type of MI table, there is some level of PU risk involved. Risk reduction and prevention measures reported included allowing patients to change positions during radiography procedures, avoiding “dragging” of patients, the use of pressure relieving aids. The need for effective collaborations with other health professionals such as nurses was also identified as a means of effectively dealing with PU risk in MI. Effective collaboration is necessary because of the specialist knowledge/skill in this area. Moreover, awareness creation and training of radiographers about PUs as well as patient centered services were also proposed. The authors acknowledge the deficiencies in knowledge on measures to prevent or reduce the risk of PU among radiographers and variability regarding radiographic procedures in the development of PUs. The sample size is small and the likelihood of type II error is high; hence, the findings of the study may not be generalisable. Justham *et al*. [13] investigated the IP experienced by healthy volunteers on MI table surfaces. The study involved 16 healthy volunteers. This study is useful because it shows the potential risk of high IPs in imaging procedures. However, it has limitations. The study calculated the mean IP of the heels, left and right buttocks, sacrum, left and right scapula, thoracic spine and occiput using the Talley Oxford Pressure Monitor™ (TPM) mark III made up of 12 cells, in 16 healthy volunteers. The TPM mark III is a pneumatic sensor pressure mapping system, made up of air cells connected to an air reservoir [20]. The cell matrix of the TPM system has poor spatial resolution due to wide spaces between sensors, some as much as 100 mm [20]. The limitation of this is that, a bony anatomical area such as the heel and the occiput may only partially cover a sensor, hence only a fraction of the IP values at these anatomical areas will be recorded. Gyi *et al*. [20] demonstrated the effects of a pressure point partially covering a sensor on the accuracy of IP readings using the TPM system which has similar spatial resolution and spacing of sensors to the one used by Justham *et al*. [13]. Gyi *et al*. [20] concluded that pressure mapping systems with poor spatial resolutions may not be reliable. This finding depicts a significant limitation in the work of Justham *et al*. [13] in that the instrument used had poor spatial resolution, with only 12 cells, which might have led to partial covering of anatomical areas, hence inaccurate IP values might have been recorded. It is therefore not surprising that the mean IP values recorded for the anatomical areas have very large standard deviations (SD), with the head and heels having the largest SDs (7.5 ± 26.2 and 7.2 ± 39.1 respectively).

Another limitation of the study conducted by Justham *et al*. [13], is that the TPM system does not record IP readings in real-time, taking an average of 12 seconds to record the data from a single scan of each of the 12 sensor matrix [20-22]. Although 12 seconds may appear to be a short time, this is a long time in terms of pressure mapping. The implication is that, it is not possible to check for errors, artefacts, and changes in a volunteer´s position, or movement during data acquisition. For example, unlike new pressure mapping technologies such as the XSENSOR™ which provides an interactive system to detect movements and artefacts the very moment they occur during pressure mapping, the TPM system may detect this movement 12 seconds after it had occurred. This will affect the IP values recorded because movement and artefacts have a direct impact on IP, and if not eliminated will invalidate the values recorded. Therefore, the IP values recorded by Justham *et al*. [13], cannot be deemed to be devoid of movement and artefact errors, hence might not be a true reflection of the IP of healthy volunteers on MI surfaces. The volunteers in the study by Justham *et al*. [13] rested their heads on a single foam filled pillow during the data acquisition and this may invalidate the results. Studies have shown that, when measuring IP of an anatomical area on a support surface, the pressure mat should be placed directly between the anatomical area under investigation and the support surface and the use of the pillow provided some level of cushioning or protection for the head and directly affecting the IP values [23-25]. Because the study investigated the IP on the X-ray table with and without mattress it is not clear why pillows were used during pressure mapping considering that it was previously stated that the use of pillows are likely to invalidate the results. Thus, the IP values recorded for the head on the imaging tables may not be a true reflection of the IP for the head on the MI tables. Also, the use of pillows may have increased the IP for the thoracic spine, sacrum, and other parts of the body because the use of a pillow is likely to elevate the head, putting more pressure on the cervical, scapulae, and thoracic spine, ultimately, increasing the IP at these anatomical areas. Therefore, the use of the pillow could result in an increased IP for these anatomical areas.

## Conclusion

There are limited studies on PUs relevant to radiography. However, the available studies provide some evidence that radiographic procedures and settings subject patients attending for radiographic procedures to an increased risk of PU development. It is imperative that further studies are conducted involving this highly specialised environment to help address the problem of the potential for PU development. Furthermore, precautionary measures should be introduced into radiography practice, to minimise the risk of PU formation and to limit exacerbating any existing PUs. It should be noted that two out of the five papers reviewed in this article were from the same author. This is a limitation for this review article and clearly indicates that there is lack of research on PUs risk in radiography. Further research on PU risk in radiography is warranted.

### What is known about this topic

- PUs poses significant threat to patients costing billions to treat and/or manage;
- The risk of medical device related PUs could occur during radiographic procedures.

### What this study adds

- This review has shown that there are limited studies on PUs relevant to radiography;
- This review helps to create the much-needed awareness of the threat of medical device related PUs in radiography;
- This review has shown that there is the need for further PU studies in radiography to help address the problem of the potential for PU development.
